# Rethinking the study of human–wildlife coexistence

**DOI:** 10.1111/cobi.13653

**Published:** 2020-10-26

**Authors:** Simon Pooley, Saloni Bhatia, Anirudhkumar Vasava

**Affiliations:** ^1^ Department of Geography Birkbeck, University of London 32 Tavistock Square London WC1H 9EZ U.K.; ^2^ School of Life Sciences University of KwaZulu‐Natal Room 105 John Bews B, Carbis Road, Scottsville Pietermaritzburg 3209 South Africa; ^3^ Centre for Technology Alternatives in Rural Areas Indian Institute of Technology Bombay Powai Mumbai 400076 India; ^4^ Voluntary Nature Conservancy 101, Radha Darshan, Behind Union Bank of India Vallabh Vidyanagar Gujarat 388120 India

**Keywords:** coexistence, human–wildlife conflict, methodology, coexistencia, conflicto humano‐fauna, metodología

## Abstract

Although coexistence with wildlife is a key goal of conservation, little is known about it or how to study it. By coexistence we mean a sustainable though dynamic state in which humans and wildlife coadapt to sharing landscapes, where human interactions with wildlife are effectively governed to ensure wildlife populations persist in socially legitimate ways that ensure tolerable risk levels. Problems that arise from current conflict‐oriented framing of human–wildlife interactions include reinforcing a human–nature dichotomy as fundamentally oppositional, suggesting coexistence requires the absence of conflict, and skewing research and management toward direct negative impacts over indirect impacts and positive aspects of living with wildlife. Human behavior toward wildlife is framed as rational calculus of costs and benefits, sidelining emotional and cultural dimensions of these interactions. Coexistence is less studied due to unfamiliarity with relevant methodologies, including qualitative methods, self‐reflexivity and ethical rigor, and constraints on funding and time. These challenges are illustrated with examples from fieldwork in India and Africa. We recommend a basic approach to case studies aimed at expanding the scope of inquiries into human–wildlife relations beyond studies of rational behavior and quantification of costs and benefits of wildlife to humans.

## Abstract (es)

Replanteamiento del Estudio de la Coexistencia Humano‐Fauna

**Resumen**

A pesar de que la coexistencia con la fauna es una meta de suma importancia para la conservación, todavía se conoce muy poco sobre ella y cómo estudiarla. Cuando hablamos de coexistencia nos referimos a un estado sustentable, pero a la vez dinámico en el cual los humanos y la fauna están coadaptados a compartir paisajes en los que las interacciones entre humanos y animales están regidas de manera efectiva para asegurar que las poblaciones de fauna persistan de maneras socialmente legítimas que aseguren niveles tolerables de riesgo. Los problemas que surgen de las actuales estructuraciones orientadas hacia la resolución de conflictos en las interacciones humano‐fauna incluyen el reforzamiento de una dicotomía humano‐naturaleza como fundamentalmente opositora, lo que sugiere que la coexistencia requiere de una ausencia del conflicto y la desviación de la investigación y el manejo hacia los impactos negativos directos por encima de los impactos indirectos y los aspectos positivos de la convivencia con la fauna. El comportamiento humano hacia la fauna está conceptualizado como un cálculo racional de costos y beneficios, haciendo a un lado las dimensiones emocionales y culturales de estas interacciones. La coexistencia está poco estudiada debido a la poca familiarización con las metodologías relevantes, incluyendo los métodos cualitativos, el rigor ético y autorreflexivo y las restricciones al financiamiento y al tiempo. Estos retos se encuentran ilustrados mediante ejemplos tomados del trabajo de campo en la India y en África. Recomendamos un enfoque básico para los estudios de caso centrado en la expansión del espectro de las indagaciones en las relaciones humano‐fauna más allá de los estudios del comportamiento racional y la cuantificación de los costos y beneficios de la fauna para los humanos.

## Introduction

Humans and wildlife are increasingly coming into contact due to climate change, habitat conversion, and species recovery and reintroductions. Thus, it is urgent to facilitate coexistence with wildlife in shared multiuse landscapes. Coexistence is, however, too seldom defined and rarely studied. In this essay, we define coexistence and then discuss why current framings of human–wildlife relations focused on conflict hinder the study of coexistence. We provide recommendations for reconceptualizing coexistence, and for studying coexistence, based on our fieldwork in India and South Africa.

Although the term *coexistence* has become more prominent in conservation science (König et al 2020), it is seldom defined (Frank et al. 2019). We favor a formulation based on Carter and Linnell (2016): a sustainable though dynamic state, where humans and wildlife coadapt to sharing landscapes and human interactions with wildlife are effectively governed to ensure wildlife populations persist in socially legitimate ways that ensure tolerable risk levels. Although developed for large carnivores, their definition applies equally to the challenges of living with large or small potentially dangerous or destructive wildlife.

Tolerance and risk are just one (important) dimension here. Coexistence does not imply there is no risk; rather, it requires tolerance of risks and the management of risks such that they remain within tolerable limits. Effective institutions and social legitimacy allow for management actions to deal with the inevitable challenges—for humans (attacks on individuals or livestock or destruction of crops or property) and wildlife (illegal killing or destruction of habitat)—when they occur (Carter & Linnell 2016).

We agree that it is necessary to “address the disparity in human norms, attitudes, and knowledge about [wildlife] among different human groups” (Carter & Linnell 2016:576). That is, in addition to employing the many instrumental and economic tools developed to respond to problematic situations, it is also necessary to “address the human and ethical facets … directly.” It is important to build trust and legitimacy and codevelop novel decision‐making processes, which take cognizance of different stakeholders’ explanatory frameworks (rational and spiritual), moral frameworks, and risk perceptions (Madden & McQuinn 2015; Lute & Gore 2019).

## Conflict as Paradigm

The primary motivation for most work on human–wildlife conflict has been protecting threatened wildlife from anthropogenic threats. These efforts have aimed to reduce the impacts on wildlife and habitats, mitigate negative impacts of wildlife, and persuade and assist locals to adapt to living alongside damage‐causing wildlife (Pooley et al. 2017). The focus has been on negative impacts of humans on wildlife and vice versa.

In their survey of the scientific literature on human–wildlife relations, Bhatia et al. (2019) found that 71% of 250 papers focused on human–wildlife conflict, 2% focused on coexistence, and 8% focused on neutral interactions. Although this study is based on a keyword search rather than the concept, the focus on conflict in the literature on human–wildlife interactions is indisputably overwhelming (König et al. 2020).

This framing positions wild animals as consciously combative with humans and reinforces a human–nature dichotomy framed as oppositional (Peterson et al. 2010). Recent thinking is more nuanced, however. For example, Bruskotter et al. (2015) conceptualize of a continuum of behaviors from intolerance to stewardship. Frank et al. (2019) propose a continuum of human responses to wildlife from conflicts to coexistence, urging researchers to consider positive as well as negative interactions. Bhatia et al. (2019) take a similar line, adding a typology of responses with the aim of better understanding the myriad factors influencing responses to wildlife. Although only 1% of surveyed papers evoked coexistence and conflict (Bhatia et al. 2019), and case studies are scarce, presumably in places where wildlife (e.g., African megafauna or rich birdlife in Indian farmlands) has survived outside protected areas both coexistence and conflicts have been present a long time.

Although Frank et al. (2019) are fully aware of the multidimensional and dynamic nature of human–wildlife interactions, their continuum framework can be interpreted as suggesting conflict and coexistence occupy opposite poles of a linear continuum. However, coexistence does not presume the absence of conflict. Conflict is a part of life, and can be a catalyst for positive change (Madden & McQuinn 2015). Another potential pitfall of the continuum concept is that as it is much easier to count direct, negative impacts than instances of coexistence, research in this shared dimension is skewed towards conflict. Counting the hits and not the misses similarly bedevils quantitative attempts to assess human–wildlife relations (Powell et al., 2020).

A review by Kansky & Knight (2014) suggests that it is indirect impacts that most shape peoples’ attitudes to local damage‐causing wildlife. A large‐scale comparative study of attitudes and behavior toward jaguars across their range (Zimmermann 2014) shows that peoples’ tolerance for damage‐causing species is often not directly related to economic damage or direct impacts and is instead influenced by sociocultural factors, including norms and attitudes toward the species. Species are persecuted, feared, revered, or protected for aesthetic, ethical, symbolic, and spiritual, as well as utilitarian and ecological reasons (Macdonald et al. 2010; Athreya et al. 2018).

## Conceptual Challenges

Striving to protect biodiversity is not the same thing as trying to promote human–wildlife coexistence, as we envision it. What is true, however, is that the study of important dimensions of coexistence (while not necessarily using the term) is not new (Treves & Karanth 2003; König et al. 2020). Research includes work on tolerance and acceptance, social science approaches (particularly psychological) to studying human perceptions, attitudes and behavior, and more inclusive and reflexive approaches to human–wildlife conflicts, as well as human–human conflicts over conservation (Treves et al. 2006; Hudenko 2012; Redpath et al. 2015).

Conceptually, the key antecedent idea is tolerance: in the area of animal behavior studies, tolerance of wildlife for humans (Whittaker & Knight 1998), and in human dimensions of wildlife research, human tolerance and intolerance of wildlife (e.g., Decker & Purdy 1988; Bruskotter & Wilson 2014). We considered overlaps and differences between tolerance and coexistence, but space does not permit a review here.

Since the early 2000s, variations on the theory of planned behavior have been used to investigate tolerance for wildlife. Bruskotter & Wilson (2014:159) concluded that behavioral intentions are the best indicators of tolerance for a species: what is meant by tolerance here is “passive acceptance of a wildlife population.” The conservation focus of concern is intolerant attitudes or judgements as the drivers of intent to harm wildlife, sometimes enacted. The aim is to reduce intolerance of wildlife hazards through framing conservation messages to address locals’ perceptions of risks and benefits, emphasizing the latter.

The focus is intolerance—and ensuing conflict between conservationists and those acting on their intolerance to wildlife—and the role of conservationists themselves is usually not considered. Tolerance (and by implication coexistence) is regarded as passive and less directly linked to action or behavior than intolerance. Seeing wildlife as a hazard suggests people formulate their judgements of the acceptability of these animals in accordance with the perceived risks and benefits associated with living alongside them. Although Bruskotter & Wilson (2014) acknowledge that this is not a completely rational process and that it includes affect and intuitive risk assessments, their focus is on decisions “driven largely by the outrage that is felt over the potential consequences” (i.e., negative impacts). Brenner & Metcalf (2019:262) define tolerance as “accepting wildlife and/or wildlife behaviors that one dislikes.”

We do not suggest this is not important and interesting work. We do suggest that the framework used is oriented to negative impacts and relationships predicated on rational calculus of costs and benefits. This line of thinking permeates recent ideas on coexistence. In a special section of this journal on human–wildlife conflict and coexistence, König et al. (2020) provide a conceptual framework with a focus on damage prevention and conflict resolution, coexistence being the goal these foci will deliver (i.e., largely absent).

The emphasis across the special section (12 articles) is mostly on reducing damages, involving relevant stakeholders in management, and providing co‐benefits calculated within a cost–benefit framework (e.g., Denninger Snyder & Rentsch 2020; Jordan et al. 2020; Treves & Santiago‐Ávila 2020). Locals are of interest mainly insofar as they perceive these costs and benefits in different ways, are affected by them in different ways, and respond to them.

In a provocative essay, Chapron and López‐Bao (2020) suggest the study of conflicts over wildlife is a distraction from the core business of conservation, recommending a focus on rights of nature, effectively excluding the study of locals’ coexistence with wildlife. While sharing their interest in indigenous ideas about the rights of nature, it is unclear to us how this fits with their desire for universal laws to defend nature's rights.

We argue that current research favors a partial view of human–animal relations focused on (and starting from) negative interactions. Although both risks and benefits are in the frame, this conception still obscures reasons for coexistence that are not directly related to the costs or benefits of living with particular wild animals. It is also better suited to scenarios involving charismatic species or those with commercial potential in iconic landscapes where economic benefits really count. This partial view focuses on conservation goals and outcomes and excludes dimensions not relevant to this framing. It also implies an overly rational and universal view of human decision‐making.

### Psychology and Decision‐Making

Possibly influenced by a much‐cited paper by St John et al. (2011), conservation scientists researching human behavior favor variations on the theory of planned behavior (Jochum et al. 2014). This theory (one among many in psychology) is built on the basic elements of attitudes, personal and social norms, and perceived control. It is oriented to rational decision‐making, as the name suggests, though variants attempt to capture the influence of emotions and prior experience too. Conservation science researchers have explored in ever increasing detail the significance of the relationships between the many variables influencing behavior (see Marchini & Macdonald [2012] for a sophisticated example). The approach is quantitative and statistical, and the aim is primarily to understand and change undesired behavior.

Research by psychologists shows that human decision‐making is not primarily or even most frequently rational, however. This is especially the case when it comes to infrequent, vivid, and traumatic events. Neurological research shows that “the emotional brain” responds to sensory inputs faster, and more simply, than the neocortex or “rational brain” (Van der Kolk 2014:60–63). In a normally functioning person, providing the stimuli from the emotional brain are not too strident, the automatic response it triggers can be evaluated and if necessary overridden by the neocortex. Traumatic events may result in posttraumatic stress disorder (PTSD), however, where the regulating function of the neocortex is disrupted and emotions and impulses related to the originating (and related) events become much harder to control. In such cases, rational explanations and facts will not silence the alarms traumatized people are receiving from their emotional brains (Van der Kolk 2014).

Hard‐won coexistence scenarios may be compromised if traumatic impacts are not recognized and addressed. Doing so is not straightforward because different cultures use different mechanisms to help those suffering from such extreme experiences (Wilson 2007). These range from the psychological and psychiatric treatments of Western societies, through mainstream religious beliefs and interventions, to healers, shamans, rituals and alternative cures used in traditional societies (and Western societies today). Humans are driven to make sense of their experiences, especially apparently random traumatic events. We worry that attempts to educate locals out of their cultural interpretations of such events (e.g., Surayawanshi et al 2013) may negate the work such beliefs do in helping people cope. What will be offered in their place?

Despite such insights from other fields, most studies of human–wildlife conflicts have been built around quantifications of damage and the effectiveness of responses intended to mitigate damage (Pooley et al. 2017). A popular strategy is to discover locals’ perceptions of damage and compare it with a quantification of damage, in the hope of convincing locals of the error of their perceptions (Surayawanshi et al 2013). Here, the truth‐telling power of the scientific method is put in direct opposition to how locals experience and explain human–wildlife relations (which include, but are not limited to, impacts). Research into PTSD suggests this is unlikely to be effective where traumatic encounters have occurred. Integrative theories focusing on how emotions and cognitions interact during decision‐making offer promising ways forward (Hudenko 2012).

Conservation scientists’ familiarity with quantification and related analyses partly explains the prevailing focus on negative impacts. Studying conflicts, and particularly coexistence, requires less familiar methodologies and interdisciplinary collaborations (Bennett et al. 2017). Therefore, while supporting current approaches to quantifying and analyzing the variables shaping human–wildlife interactions, we caution against absorbing coexistence and the kinds of influences that shape it into a quantitative, direct‐impact‐oriented framework.

The recognition that conservation scientists should collaborate with social scientists (with long traditions of studying prejudice and stereotyping and human attitudes and behavior) to address both rational and emotional dimensions of human responses to wildlife has been an important development in human–wildlife relations studies (Johansson & Karlsson 2011; St John et al. 2011; Hudenko 2012). We welcome Brenner and Metcalf's (2019) typology of attitudes to and acceptability of wildlife insofar as it suggests that tolerance and intolerance (emphasizing action or passivity and dislike) are one among several dimensions of such relations.

### Units of Study

Where should studies of human–wildlife coexistence take place, and what should they encompass? Biologists prefer to study populations and communities relatively unaffected by humans, which they regard as wild, that is, not debased through interactions with humans. They prefer to do so in wild or mostly wild landscapes. But it is precisely where humans and wild animals interact on a regular basis (as they have for millennia) that conflicts arise. It is also where coexistence is found. We agree with König et al. (2020) that conservation needs to focus on multiuse landscapes, notably agricultural areas, and be cognizant of emerging frontiers in an era of climate change, recovering wildlife (in some regions), habitat encroachment, rewilding, and emergence of zoonotic diseases.

A key challenge is to approach human–animal relations within a coherent conceptual and interdisciplinary framework, rather than splitting it into studies of human dimensions by ethnologists and of wildlife by ethologists and ecologists. We agree with Carter and Linnell (2016:575) that “coexistence emerges from the interactions” of humans and wildlife, but we are cautious about describing these as emerging “within coupled socioecological systems, in which the human and natural systems are fundamentally integrated.” This systemic conception should not obscure the individual nature of places or human and animal individuals and societies, that is, the contingency and specificities of historical, cultural, and individual behavior and interaction. Systems should be defined and subject to investigation. Coupling should not be assumed wherever the social and the environmental co‐occur, and causal links must be proven (Walters & Vayda 2009).

Our conception of coexistence assumes wild animals have the capacity to adjust to human presence in the landscape; it is not only humans who can adapt. This capacity can be individual and social. Brakes et al. (2019) and Kühl et al. (2019) make strong cases for considering animal cultures in conservation. They urge researchers to study social learning by animals and emphasize the importance of key individuals (Brakes et al. 2019:1033). Brakes et al. (2019:1033) suggest that “social learning can … be exploited to ameliorate human‐wildlife conflict,” which also suggests humans can learn much about how animals have already adapted in order to peacefully coexist with humans (and vice versa). This work offers ideas for reframing studies of human–wildlife interactions as studies of human–animal communities.

The challenge is to identify and delineate the communities of cross‐species interest—rather than focus as Brakes et al. (2019:1034) suggest on populations or social units to “predict how specific biological processes may influence conservation outcomes.” Interesting ideas for how to study human–animal communities are emerging from human–animal studies (Marvin & McHugh 2018), etho‐ethnology (Lestel et al. 2006), field philosophy (Van Dooren 2019) and multispecies ethnography (Aisher & Damodaran 2016).

Lestel et al. (2014) object to more traditional ethologists’ exclusion of animals that live or interact with humans as worthy subjects of study by deploying notions of disturbance (their term) or habituation. Dismissing anecdotes about singular animals renders ethologists unable to perceive unusual and singular animal behavior and protects their notions of typical species behavior, generalized from statistically significant sample sizes of observations. They recommend close observation of interactions between species, considering the ideas, communication styles, perceptions, and priorities of all the actors involved.

How could these diverse approaches be synthesized? A good place to start may be to establish cross‐disciplinary clarity on the terms used for human–wildlife interactions—for example, *attraction*, *habituation*, *sensitization*, *tolerance*, and *avoidance*—and to consider the value judgments attached to these terms (Whittaker & Knight 1998). *Habituation* is possibly the most misunderstood. In conservation science, major disagreements remain over whether or not habituation is good for wildlife and to what extent activities such as diversionary feeding or hazing are legitimate conservation practices (Bejder et al. 2009).

Habituation can be defined as a learning process in which repeated exposure to humans (with neither positive nor negative reinforcement) results in a reduction in response in individual wild animals because they learn there are neither costs nor benefits to the presence of humans (Bejder et al 2009). Tolerance, on the other hand, refers to the response of an individual animal in the moment (i.e., its behavioral state at a single point in time).

Studying only behavioral responses to human–wildlife interactions can mask impacts and suggest that animals showing tolerance for humans are not being negatively affected in any way. However, such animals do experience physiological responses (e.g., increased heart rate) that have no external indicators (Bejder et al. 2009; Vijayakrishnan et al. 2018). It is intriguing to speculate what might be learned by turning this around and considering similar responses in humans who have to tolerate potentially dangerous wildlife in their daily lives.

## Methodological Challenges

Perhaps as a result of their novelty in conservation science, some central tenets of good qualitative research relevant for studying coexistence are insufficiently applied. These include rigor in recording and presenting qualitative data, reflecting on the researcher's role in the research process, and thinking through research ethics. Good practice is further hindered by mismatches between academic and funding requirements and the time requirements of ethnographic‐style research.

Numerous theses and publications present qualitative data imprecisely, in ways that the same researchers would never countenance presenting quantitative data. For example, tables of interviewees that allow attribution of quotations to specific interviewees (not “an old man told me”) are seldom provided (interviews recorded rigorously as data). Transcriptions of interviews or focus groups are seldom referred to. Methods sections are silent on the social contexts in which data were collected, by whom, and how this could have influenced the information gathered.

Self‐reflexivity is an important dimension of field research, particularly research on sensitive topics such as human–wildlife conflict and coexistence. Sometimes noted, it is seldom explored in depth. In essence, it refers to researchers reflecting on their identity and how this positions them (and results in them being positioned) relative to their interviewees (Lute & Gore 2019). It reminds researchers that they bring strong biases to interview situations and that knowledge is being coproduced.

Researchers are urged to be as transparent as possible in presenting themselves and their research projects to interviewees. This is to the good, but actual field research is more complicated, raising issues around ethnicity, gender, socioeconomic status, and power relations shaping the nature of interactions (Chattopadhyay 2013). Researchers build relationships with individuals and communities that may require omitting certain aspects of their personal circumstances and beliefs, for example, religious beliefs, gender identity, sexual orientation, wealth, and access to resources (relative to interviewees).

Being invited to witness or even participate in illegal activities raises dilemmas for researchers. Researchers are not there to judge locals, but there are times when lives are at stake or their personal values are challenged, when they may feel compelled to act to prevent such activities. Participation may offer access to important knowledge but require researchers to be less judgmental. However, when it comes to writing and publication, the intimate relationships and trust that allowed participation become challenging to represent and explain, and communicating may be difficult to achieve without compromising anyone involved (Chattopadhyay 2013; Smith 2016). Self‐reflexivity and the constraints on objective observation deserve serious attention.

Researchers have an ethical duty to ensure no harm will come to those they are working with, during and after research activities (ASA 2011). Asking people to talk about traumatic events and possibly illegal responses to them requires empathy and tact. It requires putting the feelings of interviewees first when conversations become upsetting. Victims of traumatic events should be interviewed with someone close to them present to support them. Learning about peoples’ lives and experiences requires humility because researchers are the learners, not the experts. In addition to those suffering traumatic events, researchers hearing about them also have a duty of care to themselves: both sets of persons should have someone to discuss their experiences with in confidence. Finally, it is advisable to consider the sensitivities of governments and management organizations and ensure publications and public statements do not compromise local collaborators.

Time and funding constraints, especially those faced by early‐career researchers, present serious challenge for ethnographic‐style work. Postgraduate research projects and short‐term grants demand quick results and publication. Undertaking ethnographic research, however, typically takes years. It takes a brave graduate student to arrive at a field site and spend several months doing “informed hanging out” (anthropologist G. Marvin, personal communication 2018). Not doing so, however, and arriving with preformulated ideas and prestructured research instruments seriously compromises researchers’ abilities to discover concepts and questions they had not already thought of. Conceptually, ethnographic researchers try to avoid preconceptions and biases in questions and analyses based on predetermined categories and theoretical perspectives.

Studying coexistence requires slow research and a willingness and capacity to listen carefully to and learn from others. It requires researchers to take the time to approach communities appropriately, get the necessary permissions, make good contacts, win peoples’ trust, learn about their lives beyond just how they interact with wildlife, and above all empathize with them. Interviewees are individuals with unique biographies; they are not simply *victims*, *perpetrators*, or demographic variables. This work requires giving the care and attentiveness to people that naturalists give to observing nature.

It is remarkable how little attention has been paid to the experiences of (and posttraumatic effects on) people involved in life‐changing encounters with wild animals, including attacks and disastrous losses of livestock, food, or crops (Barua et al. 2013). There is a management focus on prevention and one‐off or short‐term compensation measures, but lives may be changed forever and attitudes deeply affected for the long term by such encounters.

## Fieldwork Challenges

Doing fieldwork on human–wildlife interactions brings some unusual challenges. We recommend that researchers planning research on coexistence spend time in the field first, including participant observation and open‐ended interviews. Conversations with locals ranging beyond negative interactions with wildlife may reveal unsuspected dimensions. Once researchers think they have found the right questions to ask, it is worth testing them and the potential research methods to discover factors influencing what kinds of answers their questions elicit (or fail to).

It is not sufficient to gather demographic information on, for example, gender, age, or caste without understanding how these dimensions influence social interactions, including focus groups and interviews. Caste, for example, is a complex and fluid concept that nevertheless shapes social interactions in rural India (de Zwart 2000). Access to interviewees, their exposure to particular hazards, and their risk perceptions may differ significantly by demographic group and community association (Gore & Kahler 2012). We illustrate some of these considerations through examples from our fieldwork.

Traveling around the districts of Kheda, Anand, and Vadodara in Gujarat, meeting locals known to A.V. and crocodile expert Raju Vyas from a prior survey of interactions with mugger crocodiles (*Crocodylus palustris*), the diversity of interview situations became quickly apparent. Very seldom were we alone with our select interviewee: relatives and friends would be present, and passers‐by would join in. Retaining focus on the person of interest, particularly if young or female, was challenging. If an elder or senior relative, a higher caste individual, or men (if the interviewee was a female) were present, they tended to dominate conversations and inhibit some interviewees. In some rural communities, women are reluctant to speak to unfamiliar males. It makes sense to work in a team of men and women.

Some questions, which seemed important and obvious to SP, an outsider, were met with incomprehension or evaded—in particular, direct questions to uneducated rural victims of mugger bites about how they felt about their experiences. A.V. struggled to translate and differentiate the concepts of feeling and thinking for some interviewees, a process exacerbated by variations in accents and word usages across the study region. In some cases, it remained unclear whether interviewees had suppressed their emotional responses to attacks, did not want to discuss them publicly or with strangers, or decided it was inappropriate to reveal feelings they assumed we would find unacceptable.

A 50‐year‐old man bitten near Pingal Walla village in Vadodara State and another who had lost a relative at Traj village in Kheda District said they had felt nothing toward the mugger and had just been worried about the consequences of the attacks. An elderly widow in Mahadev in Vadodara District remarked that she could not express her feelings toward the mugger that bit her because “it's illegal” (interview references in Supporting Information).

Locals from different castes interacted with us differently. Some Darbar men (warrior, ruling caste) were confident and challenging, whereas some Waghari caste (so‐called scheduled caste) were diffident. We were told that the Waghari community (mostly agricultural laborers) in a certain village were responsible for problems with crocodiles because they sacrifice animals and put the waste in the village pond. Community members denied this, as did the village *sarpanch* (mayor). In northeastern Vadodara District, we were told that scheduled castes were barred from some crocodile exclusion enclosures.

In northern KwaZulu‐Province, South Africa, S.P. interviewed a family where the adult son was killed by a leopard. The consequences of his inability to complete paying his bride price tore the family apart. His wife's family took his 2 middle children hostage until the remaining cattle were provided. It took 3 field visits to secure an informal conversation with the investigating official. He had evaded us for fear the details would be misreported in the press. Such actual and potential indirect consequences of the impacts of wildlife on humans, for victims and for others involved—which continue after the incident—are seldom explored (but see Chowdhury et al. 2016).

Narrative methods (Riessman 2008) can be useful. They focus on why and how people tell interviewers particular narratives. The content, including the veracity of the facts, may be less important than the intention. For example, a man we met living below a village pond near Heranj (who is an enthusiastic participant in mugger conservation) tried to convince S.P. that muggers presented an unacceptable danger to his family. He aimed to pressure the village *sarpanch* into building a crocodile exclusion enclosure near his homestead, thereby acknowledging his status in the community.

Conducting interviews in translation is sometimes presented as inherently compromised. That should only be the case if the researcher is not working with locals who can translate and interpret. It is a team exercise—a social activity greater than the sum of its parts—and all parties need to reflect on the process. Certainly, something will be lost, and possibly something not intended by the interviewee may be gained. Research partners knowledgeable in local customs and able to elicit responses through conversation, rather than only direct questions, are invaluable.

## An Approach to Studying Coexistence

We do not advocate studying everything; that is, the approach to knowledge described by Borges (1975) in a story about a “[m]ap of the Empire whose size was that of the Empire, and which coincided point for point with it.” We worry that mapping all the variables influencing behavior and working out the relative strengths of their interactions over time and changing circumstances may lead to the creation of just these kinds of impenetrable conceptual maps.

We suggest first delimiting case studies loosely, focusing on the observable effects of perceived conflict or coexistence and recognizing that boundaries and dimensions will change as more is learned. Second, we recommend exploring in an open‐ended way the lived realities and concerns of those affected by human–wildlife interactions (or worries about such interactions). Dimensions include histories of land use and community–conservation interactions and consideration of the lives and interactions of humans and animals.

Having identified the key concerns and motivations of those involved through a collaborative process and clearly defined what is to be described and explained, focused field research can commence. Investigations radiate out from the identified key effects of conflict and coexistence, back in time and outward in space, to reveal a widening circle of causes. Explanatory theories to be tested, including consideration of counterfactuals, emerge from the data (Walters & Vayda 2009).

**Figure 1:**
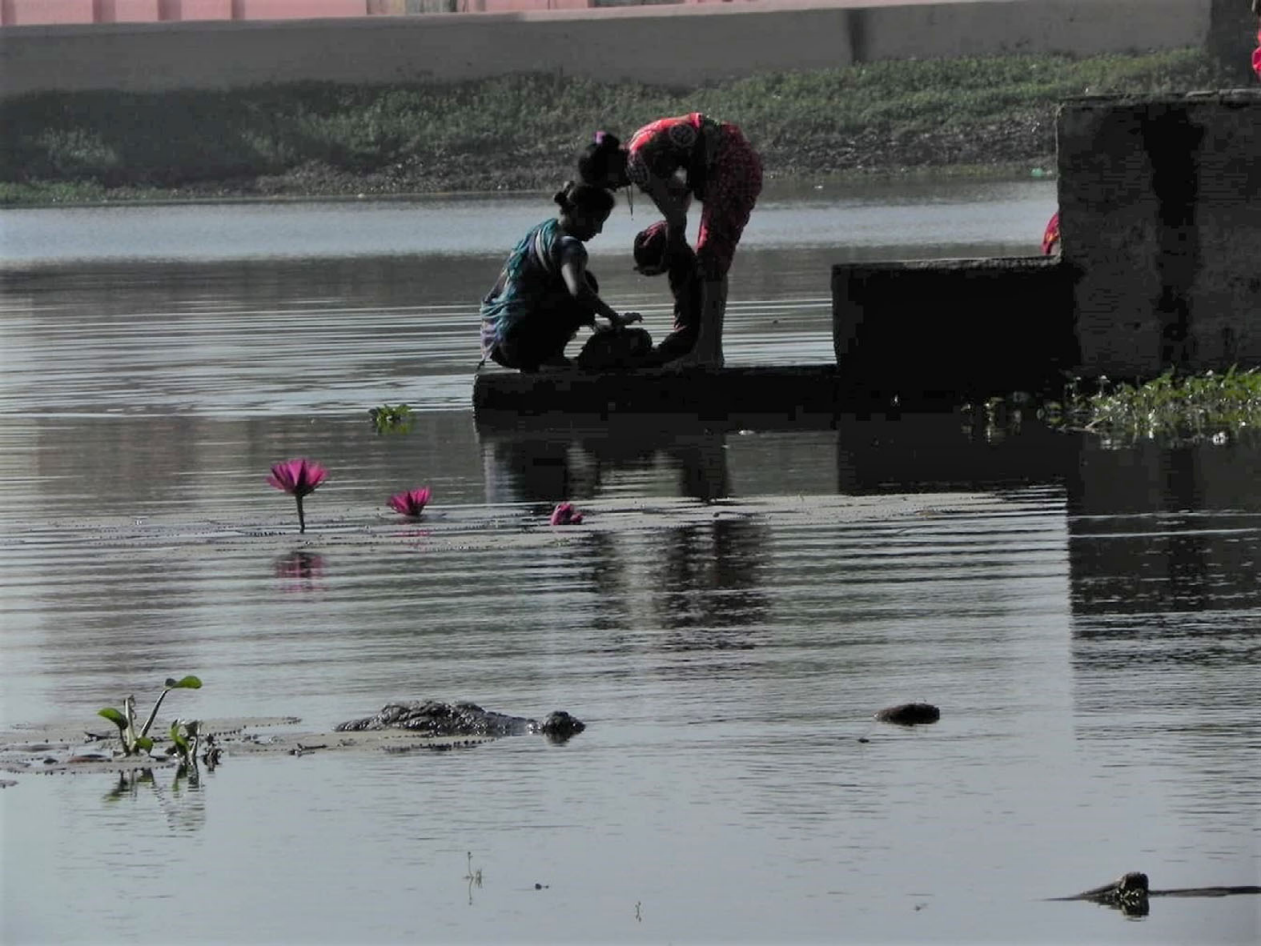
Women washing near a mugger in a village pond in Anand District, Gujarat, India.

**Figure 2:**
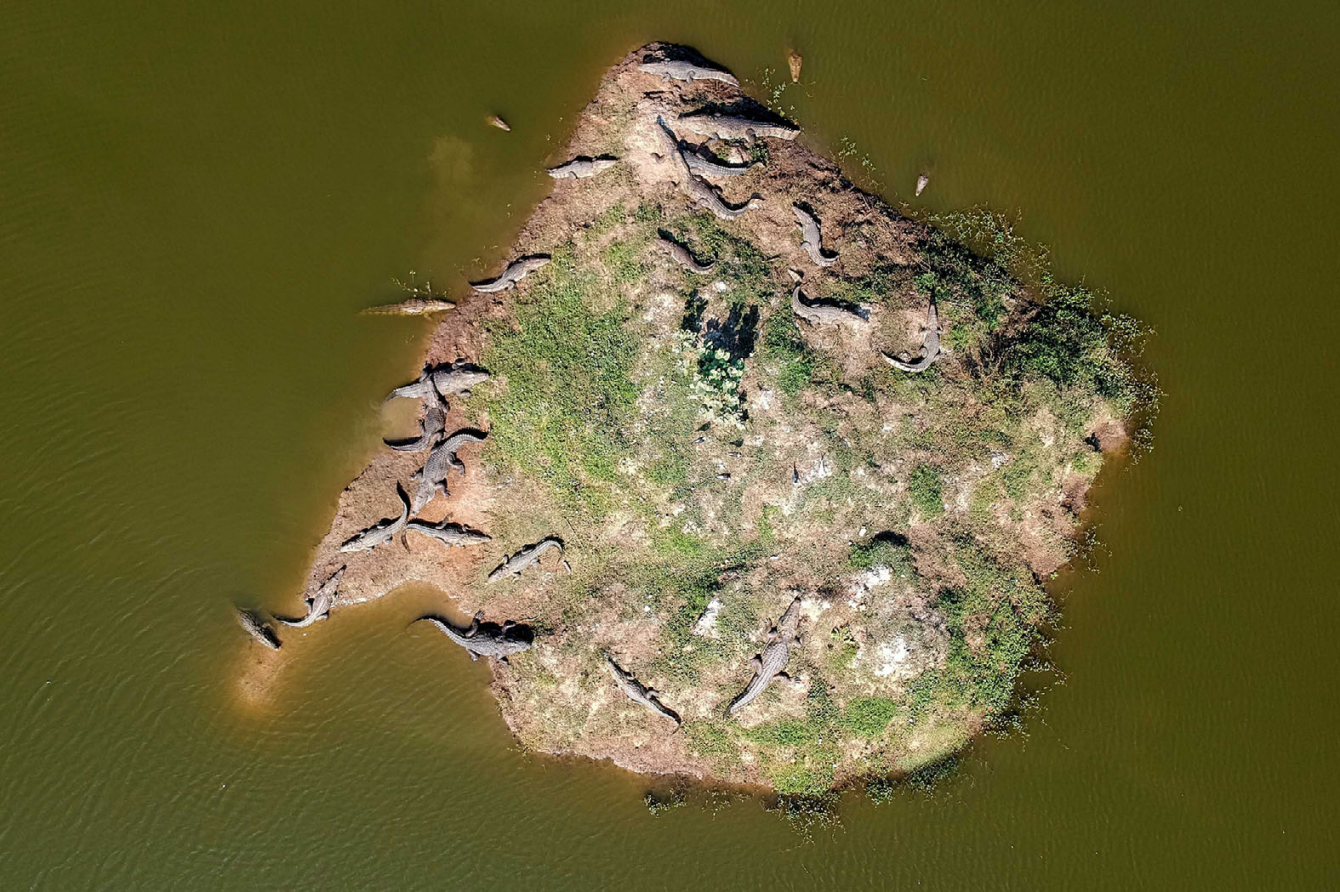
Muggers basking safely on an island created for them in Deva Village pond, Anand District, Gujarat.

Our preliminary research in central Gujarat focused on distinctive and persistent instances of coexistence (Figs. 1 & 2) and conflict with the same species: mugger crocodile. Informed by research published in Vasava et al. (2015), this involved visits to 19 villages, 3 temples, and 5 cities and towns, and numerous wetlands and rivers across 3 districts.

The following factors may influence outcomes of conflicts or coexistence regarding mugger: spiritual beliefs about nature and religious associations between the mugger and the goddess Khodiyar (variable); cultures of (and a small industry in) rescuing problem animals; levels of natural history knowledge about mugger behavior and perceptions of population trends; extent and nature of shared use of waterbodies; attacks on people or livestock; apparent differences in the behavior of muggers (mostly) resident in ponds and dams (where coexistence exists) compared with river mugger (where most conflicts arise); histories of interactions of forestry officials, locals, and nongovernmental organizations over bite incidents; and traumatic individual experiences. Comparative work on scenarios where coexistence exists and scenarios where conflicts are predominant will enable a fuller appreciation of factors influencing either outcome.

Studying coexistence where it exists, in diverse social, cultural, and ecological contexts, with appropriate methodologies and research instruments, will widen understanding of the ways in which humans and wildlife coexist. It will reveal to what degree (in particular places) coexistence requires that people do not act (show tolerance) and to what degree coexistence requires action (preventative or remedial, perhaps).

Not subordinating coexistence studies within a conception of human–wildlife interactions dominated by conflict studies, not being preoccupied with quantification of direct impacts and costs and benefits, and not universalizing Western values systems will allow exploration of novel dimensions of coexistence. These dimensions will include some not encompassed by theories of behavior focused on rational decision‐making. Historical research, human–animal studies and ethnographies, and exploring the psychology of traumatic events and the work that diverse cultural traditions do in responding to them all offer promising ways forward.

## Supporting information

Fieldwork interviews, background information, and table of interviewees (Appendix S1) is available online. The authors are solely responsible for the content and functionality of these materials. Queries (other than absence of the material) should be directed to the corresponding author.
