# Supplementary material for: Rethinking the study of human–wildlife coexistence
Source: Conserv Biol. 2020 Oct 26;35(3):784–93. doi: 10.1111/cobi.13653 (PMC8246872; doi:10.1111/cobi.13653)
Supplement: Supplementary file 1 — Fieldwork interviews, background information, and table of interviewees (Appendix S1) is available online. The authors are solely responsible for the content and functionality of these materials. Queries (other than absence of the material) should be directed to the corresponding author. [file COBI-35-784-s001.docx]

**Supplementary Information: Interviews informing essay**

This was a preliminary visit to the study region, assessing the possibilities for extended formal research. In every case, those we conversed with were informed about why we were interested in talking to them, our inability to influence management or provide material compensation for incidents discussed, their right to terminate the discussion or refuse to answer questions, and permissions were obtained to quote them (with or without anonymity as they wished). These were open-ended discussions where the only prompts were to establish details of reported events (see Vasava 2016), and responses to these events. All discussions were in the local language (Gujarati) and translating was by co-author AV. Notes were recorded in a notebook GUJ-19 by SP during interviews and subsequently digitised by SP and checked with AV.

**On the diversity of interview situations**

We (AV, SP and Vishal Mistry VM) interviewed locals in a variety of settings.

| **Date** | **Interviewee** | **Location** | **Notes** |
| --- | --- | --- | --- |
| 25/09/19 | HOTR25 | His home next to Traj village pond, Kheda District. | HOTR25 (37) and his wife were present, but only he spoke. They lost their daughter to a mugger. |
| 25/09/19 | MCTR25 | The sarpanch’s office, Traj Village | MC (36) lost his father to a mugger. He was nervous in the presence of village officials. Also present were AP (sarpanch), MP (village development officer) and VC (MC’s cousin). |
| 27.09.2019 | VGDE27 | 2^nd^ wetland of Deva Village, Anand District | VG (in his 30s) was bitten while rescuing an entangled water buffalo. Also at the interview were 7 local young men who listened in, commented, and followed us to the attack site. These included VG’s cousin GG. |
| 30/09/2019 | AJLI30 and HDLI30 | Lilod, on the Narmada River, Vadodara District | AJ is the uncle of a boy killed by a mugger. HD is the boss of local fishermen including AJ and did most of the talking. AJ’s wife and the boy’s grandmother were present but didn’t speak. Also present in our party was NP (female colleague). |

**Interviews (mentioned) related to discussion of how locals *felt* about their experiences.**

| **Date** | **Interviewee** | **Location** | **Notes** |
| --- | --- | --- | --- |
| 28/09/19 | RVPW28 | Pingal Wada village, Vadodara District | Present were RV (50), his uncle, another male relative, and his wife; in our party SP, AV, Raju Vyas (RV). |
| 25/09/19 | MCTR25 | The sarpanch’s office, Traj Village | See table above. |
| 29/09/2019 | MNVMP29 | Her home in the tiny village of Mahadev Pura, Vadodara District | MNV, an elderly widow who lives alone, was supported by the wife of a younger brother of her husband’s. Also present were AV, SP and RV. |

**On different castes**

| **Date** | **Interviewee** | **Location** | **Notes** |
| --- | --- | --- | --- |
| 25/09/19 | MCTR25 | The sarpanch’s office, Traj Village | MC was diffident in the presence of forward caste town officials. |
| 30/09/2019 | PMK30, and PSJK30 | Kanjetha Village, on the Narmada River, Vadodara District | Family of victim (10-year-old girl) had left the area. We spoke with the local priest, and PSJ joined in. He is warrior caste, very confident and challenging. Also present, 5 other locals joined; SP, AV, VM, NP |
| 27.09.2019 | VGDE27 | 2^nd^ wetland of Deva Village, Anand District | VGDE27 is Waghari community |

**With the regard to why people tell you the narratives they do:**

| **Date** | **Interviewee** | **Location** | **Notes** |
| --- | --- | --- | --- |
| 27/09/2019 | NTHE27 | Road next to his homestead just outside Heraj Village | We interviewed NT in our truck in a rainstorm, between the village pond and his home. Present were SP, AV, and in the back, VM and driver Mehul Patel (MP). |

**Interview relating to killing of a local man by a leopard, KwaZulu-Natal, South Africa.**

| **Date** | **Interviewees** | **Location** | **Notes** |
| --- | --- | --- | --- |
| 9/01/2019 | Mzileni family | Homestead near Jozini, KwaZulu-Natal Province, S. Africa | Present were the parents of the man killed by a leopard), his elder brother and the victim’s eldest daughter (18). Also SP, Abednigo Nzuza (translating) and Satchile Mzileni who introduced us to the family. |
| 10/01/2019 | IOTL19 | Jozini Tiger Lodge | Interviewee was an investigator and requested anonymity, handwritten notes only taken (minimised). |
